# Study about Mechanical Property and Machinability of Polyimide

**DOI:** 10.3390/polym10020173

**Published:** 2018-02-11

**Authors:** Shijun Ji, Jilong Yang, Ji Zhao, Yanjuan Hu, Hong Gao

**Affiliations:** 1 School of Mechanical Science and Engineering, Jilin University, Changchun 130025, China; jishijun@jlu.edu.cn (S.J.); jilong17@mails.jlu.edu.cn (J.Y.); jzhao@jlu.edu.cn (J.Z.); 2 College of Biological and Agricultural Engineering, Jilin University, Changchun 130025, China; 3 School of Mechanical and Electrical Engineering, Changchun University of Technology, Changchun 130012, China

**Keywords:** polyimide (PI), mechanical property, machinability, nano-indentation, single point diamond turning (SPDT)

## Abstract

Polyimide (PI) is a kind of polymer material with properties of high heat-resistance and good mechanical strength. As a special engineering material, it has been widely used in the fields of aviation, nanotechnology, etc. PI has been regarded as one of the most promising engineering plastics in the future. Therefore, further research must be made on its mechanical properties and machinability of the PI, especially in ultra-precision machining. In this paper, both of the mechanical properties and machinability have been studied respectively. Through the nanoindentation experiment, the nanoindentation hardness and elastic modulus of PI are analyzed. Also, the single point diamond turning (SPDT) experiment is conducted to show that the form accuracy and surface roughness of PI surface can reach a submicron degree in peak-to-valley (PV) and a nanometer scale in surface roughness (*R*a) respectively. The results demonstrate that the PI possesses good mechanical properties and machinability.

## 1. Introduction

As one of the most promising high-performance materials (HPM), the PI is known for its excellent mechanical and electrical properties, outstanding thermal stability, superior chemical resistance, and high hardness [1,2,3,4]. Owing to those distinctive properties, PI has been widely used in the fields of aviation, aerospace, micro-electronics nanotechnology, etc. [5]. It has been regarded as one of the most promising engineering materials in the future.

The excellent properties of PI make it a metal substitution in some special areas, where the machining precision is particularly important [6,7]. PI is commonly manufactured by traditional methods of molding and processing for thermoplastic—such as extrusion molding, injection molding and compression molding—which cannot achieve high surface precision. Therefore, the machined workpieces need additional post-processing to reach the high surface roughness and form accuracy [8,9]. Among those existing ultra-precision machining methods, the single point diamond turning (SPDT) process is outstanding due to the superior advantage of generating higher surface quality with only one processing step [10,11,12]. SPDT can produce components with micrometer to sub-micrometer form accuracy and surface roughness in the nanometer range [13]. So, it can be used to assess the machinability of PI in micro scale for this study.

**Figure 1:**
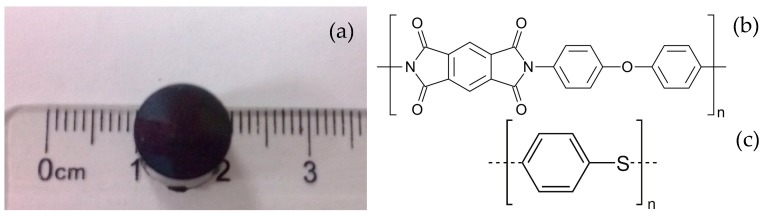
The sample used in the experiment: (**a**) PI material sample; (**b**) SP-1 structure; (**c**) PPSCA30 structure.

In this paper, both of the microscopic mechanical property and machinability have been studied, respectively. Through the nanoindentation experiment, the hardness and elastic modulus of PI are analyzed. Then, the SPDT experiment is conducted to show its excellent machinability. To evaluate the potential of PI materials better, PI materials (DuPont Vespel SP-1, Curbell Plastics, Wilmington, DE, USA) and PPSCA30 are used to do the SPDT experiment, and their structures are shown in Figure 1.

## 2. Nano-Indentation Experiments and Results Analysis

Nanoindentation is a powerful and advanced way to measure mechanical properties such as Young’s modulus and hardness of various materials. This method has been widely used to study the mechanical properties of polymers and nanocomposite [14,15,16,17]. DuPont Vespel SP-1 parts and shapes are specified for their excellent physical properties, including electrical and thermal insulation at high temperatures. In the nanoindentation experiment, several cylindrical samples produced by the method of extrusion are made of the SP-1, which is shown in Figure 1a.

**Figure 2:**
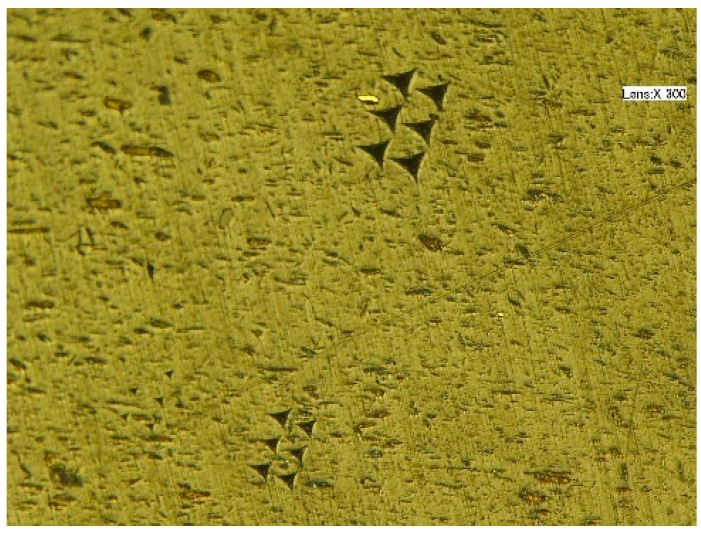
The indentation points of PI under the microscope.

The mechanical properties of PI are investigated in a micro view. Therefore, a nanoindentation apparatus (Agilent Technologies, Santa Clara, CA, USA) is needed to conduct the experiments. Here, the indenter has a triangular pyramid tip with 120° apex angle and 1.2 mm diameter. To better explore how the nanoindentation hardness and elastic modulus change in different indentation depth, five groups of experiments are designed with indentation depth of 0.5, 1, 2, 4, and 6 μm respectively. To make the experiments more persuasive, six points in different sites on the samples are tested in each group with the same indentation depth. Because the mechanical properties of PI depend on the temperature, all experiments are conducted in a constant temperature precision laboratory whose temperature is 20 ± 1 °C. The indentation points of PI under the microscope is shown in Figure 2.

**Figure 3:**
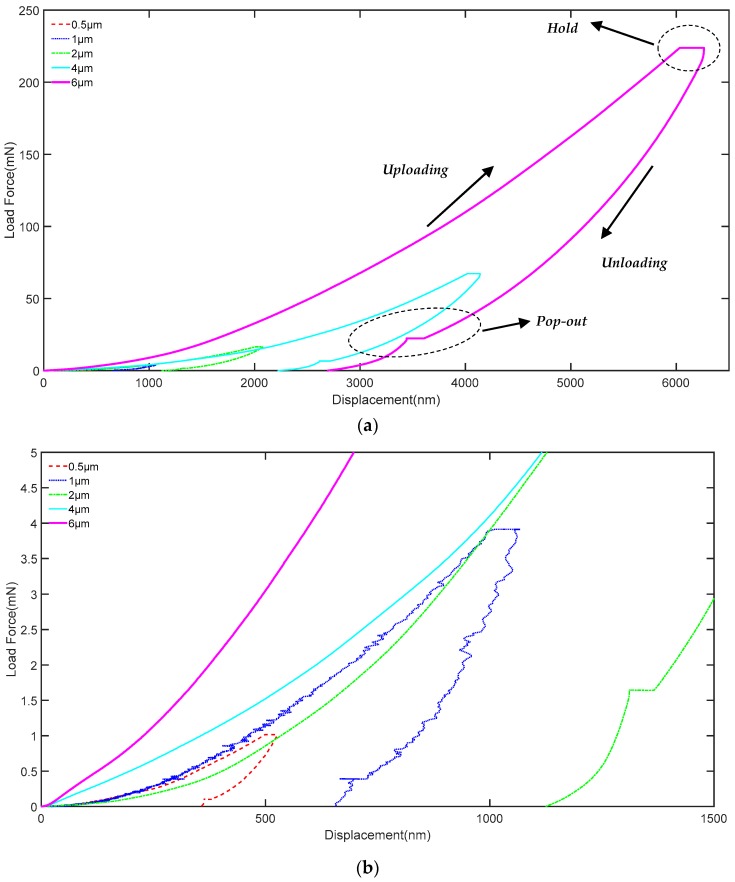
The load-displacement curve of PI: (**a**) is the load-displacement curve of PI; (**b**) is drawing of partial enlargement.

During the nanoindentation experiment, the load and displacement will be continuously recorded to form a series of relationship curves, which can better demonstrate the mechanical properties of the materials. Here, the load-displacement curves in different maximum pre-setting indentation depth are shown in Figure 3.

From Figure 3, it can be seen that five groups of load-displacement curves in different indentation depth with similar variation trend are given. The indentation depth increases smoothly with the increase of the load force in the uploading process. However, it is not a linear increase as the plastic deformation existing. During the holding time, although the load no longer changes, the displacement also keeps increasing. Here, the increased depth depends on the different maximum pre-setting indentation depths. During the unloading process, all the curves have a pop-out phenomenon, which means the existence of creep deformation [18]. The load-displacement curves in the nanoindentation experiment present a better uniformity that demonstrates the PI possesses superior micromechanical properties.

The Agilent G200 applied in the experiment can also provide the nanoindentation hardness and elastic modulus values, which are the two main parameters of the mechanical properties for PI. When approaching the designed depth, the peak hold time is 2 s. The values of those two parameters in six different points for each indentation depth are displayed in Table 1 and Table 2 respectively, accompanied by the mean values.

From Table 1 and Table 2, it can be seen that, for every designed indentation depth in the nanoindentation experiment, there are six test points with the hardness and elastic modulus value. Those tested value are variable around an average value, which indicates that the mechanical property of PI material is stable. Here, a fact must be declared that the actual depth cannot be completely identical with the designed depth due to the displacement error in the motion direction of the triangular pyramid head.

**Figure 4:**
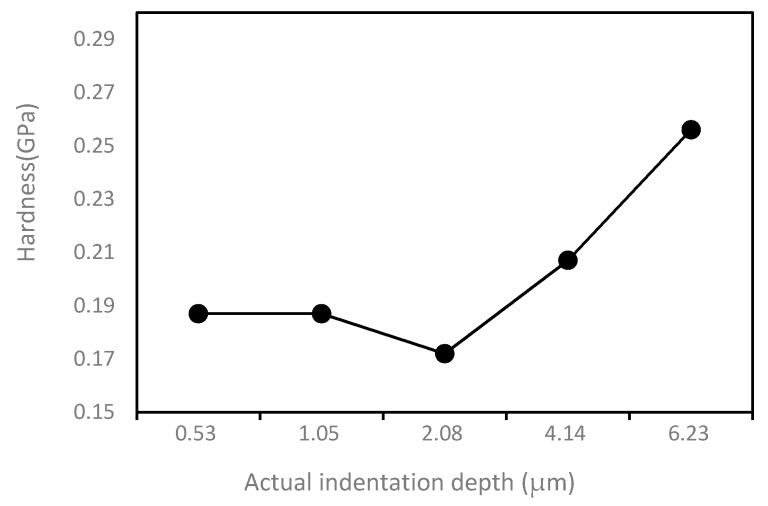
The relationship between nanoindentation hardness and indentation depth.

**Figure 5:**
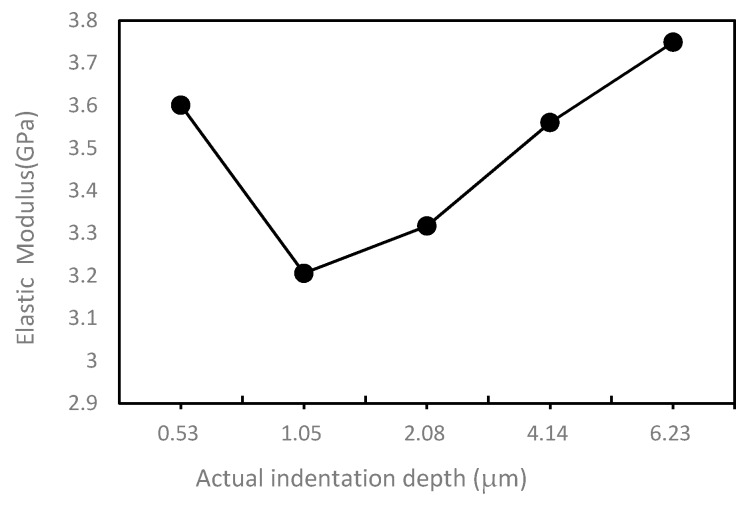
The relationship between elastic modulus and indentation depth.

To better analyze the relationship between two parameters and indentation depth, two curves obtained from two tables are also given, which are shown in Figure 4 and Figure 5. In the Figure 4, it can be observed that the hardness value fluctuates in a very small range from 0.172 to 0.256 GPa, which means that the PI has stable mechanical properties. The hardness value decreases first and then increases with the increase of the displacement. The relationship between elastic modulus and indentation depth is shown in Figure 5. The elastic modulus should have a similar trend with the indentation depth-hardness curve, but the Figure 5 shows that it has an irregular variation with the increasing of indentation depth, which may be caused by the discontinuity of the PI material. The reason why the PI material is discontinuous may be that the solidification mechanism of the surface layer is different from that of the inner layer. During extrusion, the solidification rate of the cortical layer is faster, while the solidification rate of the core layer is slower. When the cortex has solidified, the core layer is still in the viscous flow condition. The structure of core layer is looser, and the structure of microcrystal is coarse and disorderly, which leads to the different properties between cortical layer and core layer.

## 3. SPDT Experiments and Results Analysis

**Figure 6:**
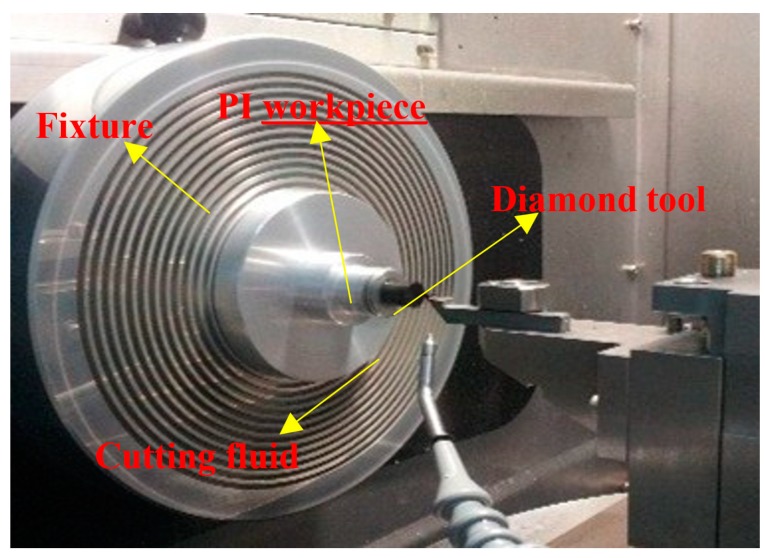
The main section of the SPDT setup.

PI has been widely used in some special fields, especially in the ultra-precision machining field. So, how to find a machining method that can achieve high surface quality seems to be particularly important. In this paper, SPDT machining technology will be applied to further investigate the machinability of PI. Here, Figure 6 displays a photograph of the main section of the SPDT experiment setup.

The SPDT experiment is conducted on a two-axis (*x*-axis and *z*-axis) ultra-precision lathe NANOFORM 250 (Precitech, Keene, NH, USA). During the cutting process, the PI workpiece is installed on the spindle through the air chuck and rotated with the spindle at a certain speed. The different cutting depth and speed can be achieved through the movement of *x*-axis and *z*-axis. Here, some machining parameters have *S* = 1500 rpm and *F* = 1 mm/min. A single-crystal diamond cutting tool used and some parameters of the tool are also given, 0.5 nm radius, 0° rake angle, and 10° clearance angle.

**Figure 7:**
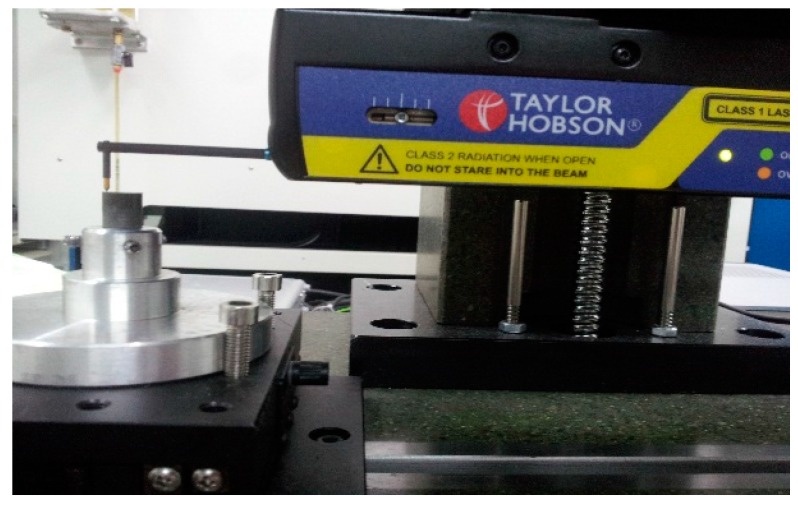
The schematic diagram of measurement.

In this SPDT experiment, a simple flat surface of the PI is machined, which is measured to see whether the ultra-precision machining is a feasible machining method for PI to have an ultra-precision surface. Here, a high-precision commercial instrument TAYLOR HOBSON (Ametek, Leicester, UK) is applied to measure the form accuracy and surface roughness of the machined surface through a contact probe of the measurement instrument. The measuring process is given in Figure 7.

**Figure 8:**
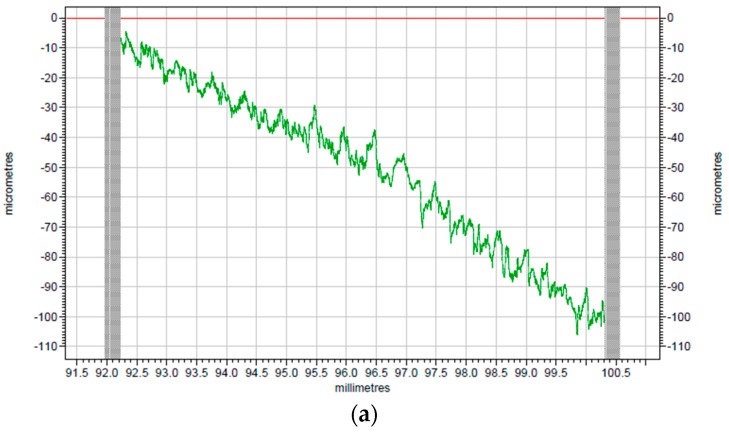

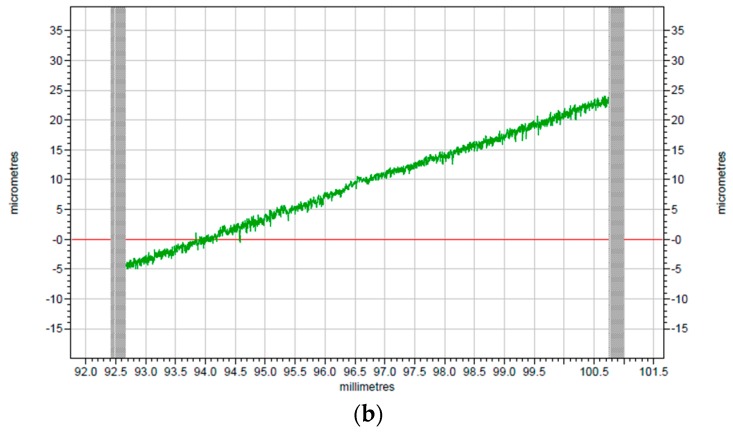
The surface profiles: (**a**) surface before turning; (**b**) surface after turning.

To better investigate the cutting effect, the comparison between raw profiles of surface before and after turning provided by TAYLOR HOBSON is given in Figure 8a,b. As indicated in Figure 8, the smoothness of the machined surface has been greatly improved when compared with the original surface.

**Figure 9:**
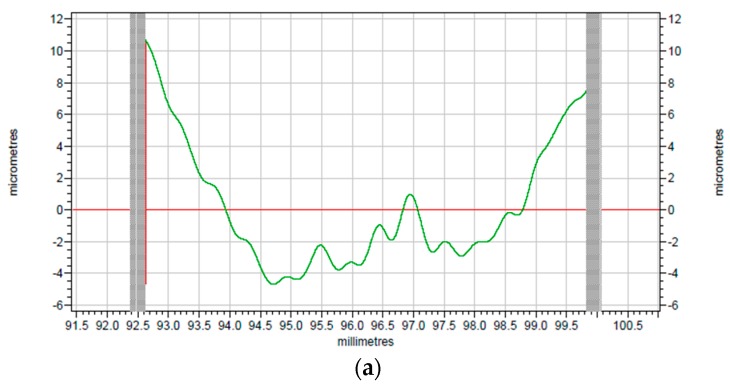

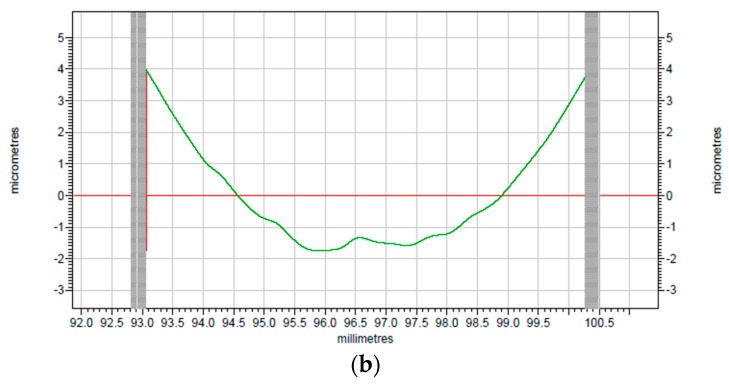
The form accuracy: (**a**) surface before turning; (**b**) surface after turning.

**Figure 10:**
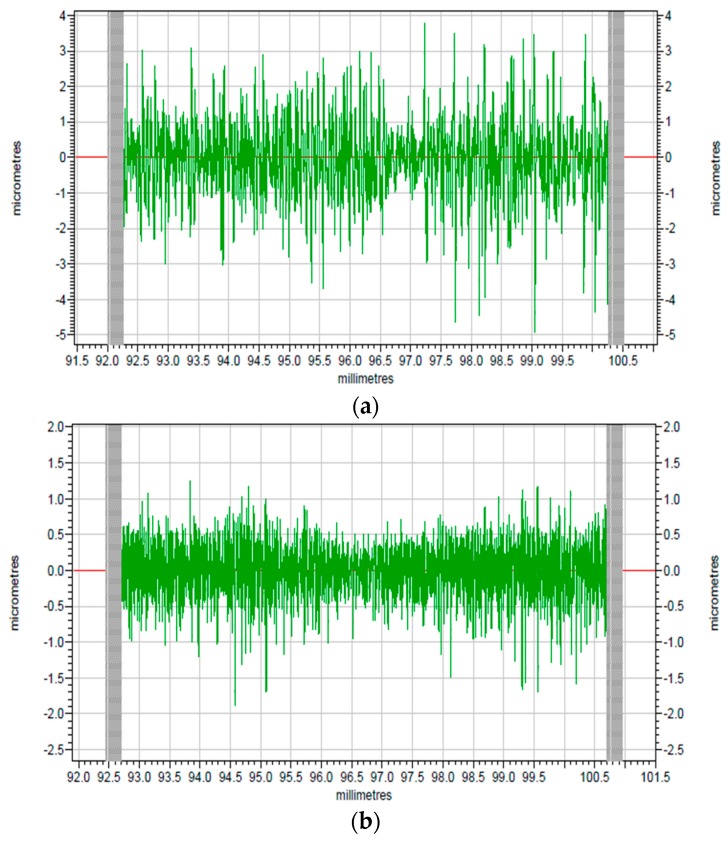
The surface roughness: (**a**) surface before turning; (**b**) surface after turning.

**Figure 11:**
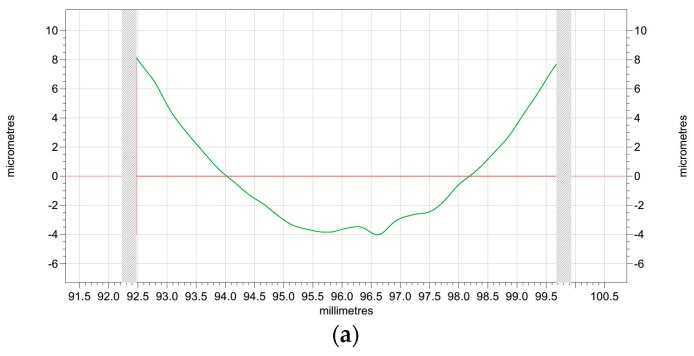

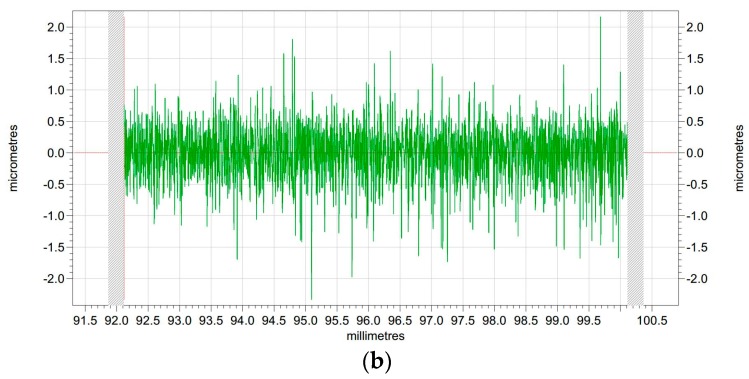
The measurement results of PPSCA30: (**a**) the PV value; (**b**) the *R*a value.

The corresponding modified profiles of the raw profiles are shown in Figure 9 and Figure 10, which are form accuracy and surface roughness respectively. The peak-to-valley (PV) value is used to evaluate the form accuracy of the machined surface. As shown in Figure 9a, the PV value of the original surface is 15.38 μm, while the machined PI surface is 5.72 μm according to Figure 9b, which has a 9.66 μm drop when it is compared with the surface before turning. Thus, the surface roughness shown in Figure 10 can be analyzed in the same way. The surface roughness (*R*a) of the original surface shown in Figure 10a is 0.758 μm, while after SPDT, it drops to only 0.254 μm as shown in Figure 10b. Because all the former processing conditions of Figure 10a are the same as the processing conditions of Figure 10b, the *R*a value and the PV value are similar to those of PI. After SPDT experiment, the *R*a value of PPSCA30 is 0.297 μm, and the PV value of PPSCA30 is 12.17 μm, which are shown in Figure 11a, b. The *R*a value of PI is 0.043 μm smaller than the *R*a value of PPSCA30, and the PV value of PI is 6.45 μm smaller than the PV value of PPSCA30. From the results discussed above, it can be seen that the PI material has a better surface quality when machined by SPDT, which means that the ultra-precision machining strategy SPDT is applicable for PI material to generate a high-quality surface and the machinability of PI is excellent.

## 4. Conclusions

In this paper, both of the nanoindentation experiments and SPDT experiments have been conducted to demonstrate the microscope mechanical property and machinability of the polymer material PI. In the nanoindentation experiments, the nanoindentation hardness and elastic modulus at different indentation depths are analyzed, which shows that the microscope mechanical property of PI is good. Also, a flat surface of PI is fabricated in the SPDT experiments and two important parameters are obtained to investigate the machinability of PI in ultra-precision machining. The form accuracy in PV and the surface roughness in *R*a indicate that the PI also possesses an excellent machinability.

## Figures and Tables

**Table 1:** Nano-indentation hardness value of each indentation point (GPa).

Designed Depth	Hardness						Mean Hardness	Mean Actual Depth
	Point 1	Point 2	Point 3	Point 4	Point 5	Point 6		
0.5 μm	0.197	0.209	0.102	0.21	0.214	0.191	0.187	0.527 μm
1 μm	0.178	0.195	0.179	0.22	0.202	0.148	0.187	1.054 μm
2 μm	0.152	0.162	0.143	0.208	0.193	0.176	0.172	2.079 μm
4 μm	0.198	0.229	0.208	0.234	0.217	0.153	0.207	4.135 μm
6 μm	0.196	0.187	0.243	0.23	0.328	0.351	0.256	6.233 μm

**Table 2:** Elastic modulus value of each indentation point (GPa).

Designed Depth	Modulus						Mean Modulus	Mean Actual Depth
	Point 1	Point 2	Point 3	Point 4	Point 5	Point 6		
0.5 μm	3.524	4.53	2.29	3.823	3.505	3.933	3.601	0.527 μm
1 μm	3.331	3.272	3.314	3.494	2.993	2.833	3.206	1.054 μm
2 μm	2.984	3.496	2.77	3.718	3.531	3.405	3.317	2.079 μm
4 μm	3.387	3.942	3.601	3.751	3.697	2.98	3.56	4.135 μm
6 μm	3.367	3.255	3.731	3.485	4.257	4.399	3.749	6.233 μm
